# Comparison of CT-perfusion software packages and validation with true infarct core in patients with acute ischemic stroke

**DOI:** 10.1186/s12880-025-02002-7

**Published:** 2025-12-02

**Authors:** Maximilian Thormann, Maria Faltass, Roland Schwab, Stefan Klebingat, Daniel Behme

**Affiliations:** 1 Department of Nuclear Medicine, Charité Berlin, Berlin, Germany; 2 Department of Neuroradiology, University Hospital Magdeburg, Leipziger Str. 44, 39120 Magdeburg, Germany; 3 STIMULATE Research Campus Magdeburg, Magdeburg, Germany

**Keywords:** Computed tomography perfusion, Acute ischemic stroke, Mechanical thrombectomy

## Abstract

**Background:**

Computed tomography perfusion (CTP) is widely used to evaluate acute ischemic stroke (AIS) and guide endovascular thrombectomy (MT). However, substantial variability exists among software solutions in estimating ischemic core volume, potentially affecting patient selection and treatment outcomes.

**Materials and methods:**

We retrospectively analyzed 65 patients with M1/M2 occlusions who underwent successful MT (TICI 2c/3). All patients had baseline CTP and follow-up non-contrast CT (ncCCT) within 10–25 h. Final infarct volumes were segmented on ncCCT. CTP data were processed using syngo.via (Siemens Healthcare) with three settings (A: CBV < 1.2 ml/100 ml; B: same threshold plus smoothing; C: rCBF < 30%) and Cercare Medical Neurosuite (CMN, Cercare Medical). We compared software-derived core volumes to ncCCT -measured volumes using Wilcoxon-Signed-Rank tests, Bland–Altman analyses, and intraclass correlation coefficients.

**Results:**

CMN showed the smallest median error (0.0 ml) and a 50.0% rate of overestimation. Among syngo.via settings, method B had improved agreement but still tended to overestimate infarct volume in larger strokes. Settings A and C more frequently produced substantial overestimations. Bland–Altman plots demonstrated that deviations from true infarct volumes increased with larger cores for all software packages, underscoring the challenge of accurately quantifying extensive ischemic lesions.

**Conclusion:**

Our findings reveal marked variability in core volume estimates across different CTP software solutions. CMN and syngo.via B provided good accuracy, but performance declined with larger infarcts. Awareness of these discrepancies is critical for clinicians interpreting perfusion maps to optimize AIS treatment decisions and avoid misclassification of patients who might still benefit from reperfusion therapy.

## Introduction

Computed tomography perfusion (CTP) is commonly used to evaluate patients with acute ischemic stroke (AIS) for endovascular thrombectomy (EVT) [1–4]. CTP offers rapid assessment of cerebral perfusion deficits and ischemic core size. In the DAWN and DEFUSE trials the use of CTP broadened the paradigm of care for patients presenting in the late window [5, 6]. Current guidelines advocate perfusion imaging primarily for patients presenting beyond 6 h after stroke onset or in select situations as an adjunct to standard imaging [7]. The primary objective of perfusion imaging in AIS patients is to identify those with areas of critical hypoperfusion. This is typically estimated using a relative cerebral blood flow (rCBF) threshold of less than 30% compared to normally perfused brain tissue or using a cerebral blood volume (CBV) threshold [4]. Distinguishing the ischemic core from the surrounding penumbra is essential for optimizing the treatment strategy in patients with AIS.

When combined with unenhanced imaging, perfusion imaging can aid in the diagnosis and triage of patients with AIS in several ways: It allows for the detection of distal and posterior circulation vessel steno-occlusive disease using Tmax maps [4, 8–10]. It can also be of use in identifying patients with a large area of established infarction who might still be suitable for EVT if a substantial portion of tissue remains at risk [11]. Perfusion imaging can also help pinpoint the symptomatic site in cases of multiple intracranial occlusions or stenoses [12]. Thus, the additional data from CTP can aid in procedural planning and help navigate more nuanced risk-benefit scenarios, particularly in patients with moderate to large core infarcts, distal vessel occlusions, or significant comorbidities [4].

Perfusion imaging post-processing is becoming increasingly automated, providing volumetric outputs within minutes of image acquisition [4]. However, the outputs of different software packages can vary significantly, and inter-vendor variability contribute to relevant differences in perfusion results [4, 13–15]. The extent to which different software solutions can be used interchangeably to predict the infarct core remains a source of confusion for clinicians [2, 16]. It is therefore advisable to use software validated on clinical datasets, where the clinical relevance of the estimated volumes has been well established [17].

The objective of this study was to compare ischemic stroke core volumes derived from two commonly used perfusion softwares, syngo.via (Siemens Healthcare, Erlangen, Germany) and Cercare Medical Neurosuite (Cercare Medical, Aarhus, Denmark), a newly developed automated CTP analysis package. Perfusion results were compared to the true infarct volume segmented on non-contast CCT (ncCCT). To attain consistency in results, only patients who underwent mechanical thrombectomy (MT) with satisfying results (TICI 2c and 3) were included.

## Material & methods

### Study population

In this single-center, retrospective analysis we included all consecutive acute stroke patients admitted to a level I university clinic in Germany between January 2020 and November 2022.

The inclusion criteria were:

- Clinical diagnosis of acute ischemic stroke.
- Confirmed intracranial large artery occlusion of the middle cerebral artery (M1 or M2).
- CT Perfusion dataset available.
- Patient underwent MT with a TICI 2c or 3 result.
- Available ncCCT within 36 h.

Exclusion criteria were:

- Severe intracranial hemorrhage on CCT.
- Severe motion artifacts or poor scan quality on initial or follow-up CCT.
- Failed automated perfusion calculation.

During the selected period, a total of 853 patients available CTP datasets. We excluded 763 patients who did not have a MCA M1/M2 occlusion, did not receive MT or did not have an angiographic TICI 2c/3 result. Another 25 patients were excluded for imaging artifacts or severe intracranial bleeding.

**Fig. 1:**
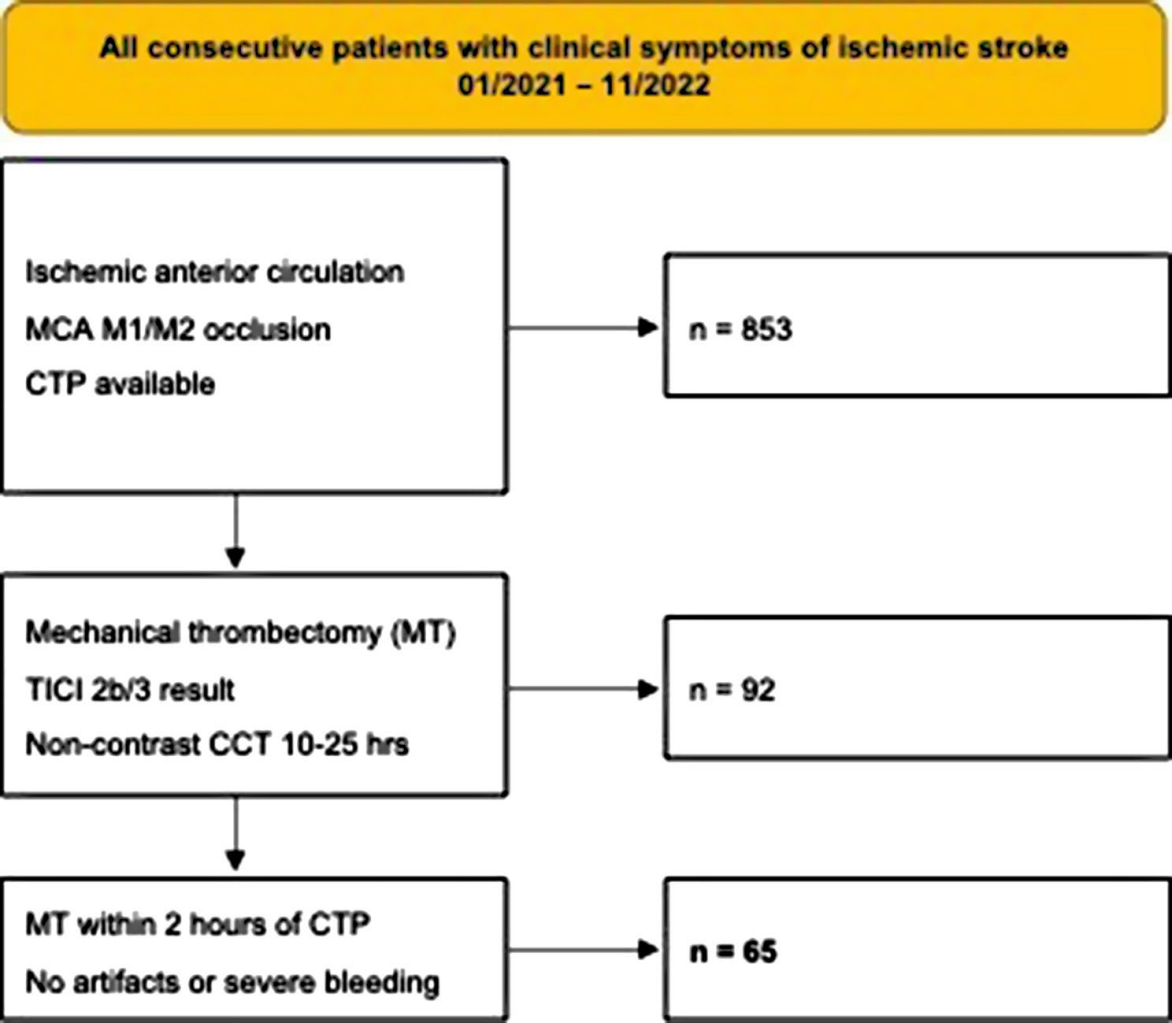
Pathway for patient inclusion

Our search identified 65 patients for inclusion (Fig. 1).

The study was approved by the local ethics committee (Nr. 11/22) and conducted in accordance with the Declaration of Helsinki.

### Imaging acquisition and post-processing

All CTP-scans were performed on the same scanner type (Somatom Definition AS+ (Siemens Healthcare, Germany). Imaging parameters were: Kernel T20F; acquisition time: 225426 ms; contrast agent: Imeron 300 (Bracco Imaging, Germany); injection rate 5 ml/s, start of acquisition: 3 s after injection.

All perfusion data were post-processed using two fully automated software packages: Syngo.via CT Neuro Perfusion (version VB60A) and Cercare Medical Neurosuite (version CMN 15.0). All software packages apply automated registration, segmentation, and motion correction.

Reperfusion after MT was assessed using the modified Thrombolysis in Cerebral Infarction (mTICI) scale score. Scoring on all post-MT angiograms was performed by an experienced neuroradiologist with more than ten years of experience (D.B.).

Syngo.via CT Neuro Perfusion uses a delay-insensitive deconvolution model and interhemispheric comparison. It identifies the lesion side by the highest time-to-drain, using the opposite side as a reference for relative values. Summary maps highlight the ischemic core and its volumes.

Adopting the approach by Koopman et al., analysis with syngo.via was done with three different settings [1]. In method A maps were generated based on default software settings. Ischemic core volume was defined as a CBV < 1.2 ml/100 ml. In method B an additional smoothing filter was added to the same threshold. In method C ischemic core was defined as a reduction of CBF < 30% ompared with healthy brain tissue. We included the CBV-based definition to test the software’s default approach alongside the rCBF criterion, which is commonly used in current practice.

Cercare Medical Neurosuite (CMN) is a rather recent addition to the market. The software adopts a model-based approach to quantify cerebral blood flow (CBF). CMN uses a model-based perfusion analysis. Instead of standard SVD deconvolution, it employs a gamma-variate model of the tissue residue function to estimate CBF. This approach, incorporating elements of AI-driven modeling, is intended to better capture prolonged contrast transit times and improve core estimation in low-flow regions.

### Clinical data collection

All case specific and demographic data were taken from the hospital information system.

### Follow-up imaging

All patients underwent follow-up using a ncCCT between 10 and 25 h after endovascular thrombectomy. The time of follow-up was at the discretion of the attending neurologist and neuroradiologist. Final infarct volume was determined using ITK snap, an open-source and multi-platform 3D medical image analysis software [18]. Briefly, to segment the image, the 1 mm slice series were uploaded to the software. The hypodense area marking the infarct was semi-automatically segmented in the axial plane. Contours were manually adjusted where necessary. Baseline CTP core measurements were compared with final infarct volumes as determined on ncCCT.

### Statistical analysis

Data were presented as mean +/- standard deviation, median (interquartile range (IRQ)) and number (percentage), as appropriate. The Wilcoxon test for paired differences was used as a non-parametric test. Bland–Altman analyses with 90% limits of agreement and calculation of intraclass correlation coefficient (ICC) (two-way mixed model for absolute agreement, single measure) were performed to determine agreement between the true infarct volume and perfusion outputs for each setting. P values < 0.05 were considered statistically significant.

## Results

**Fig. 2:**
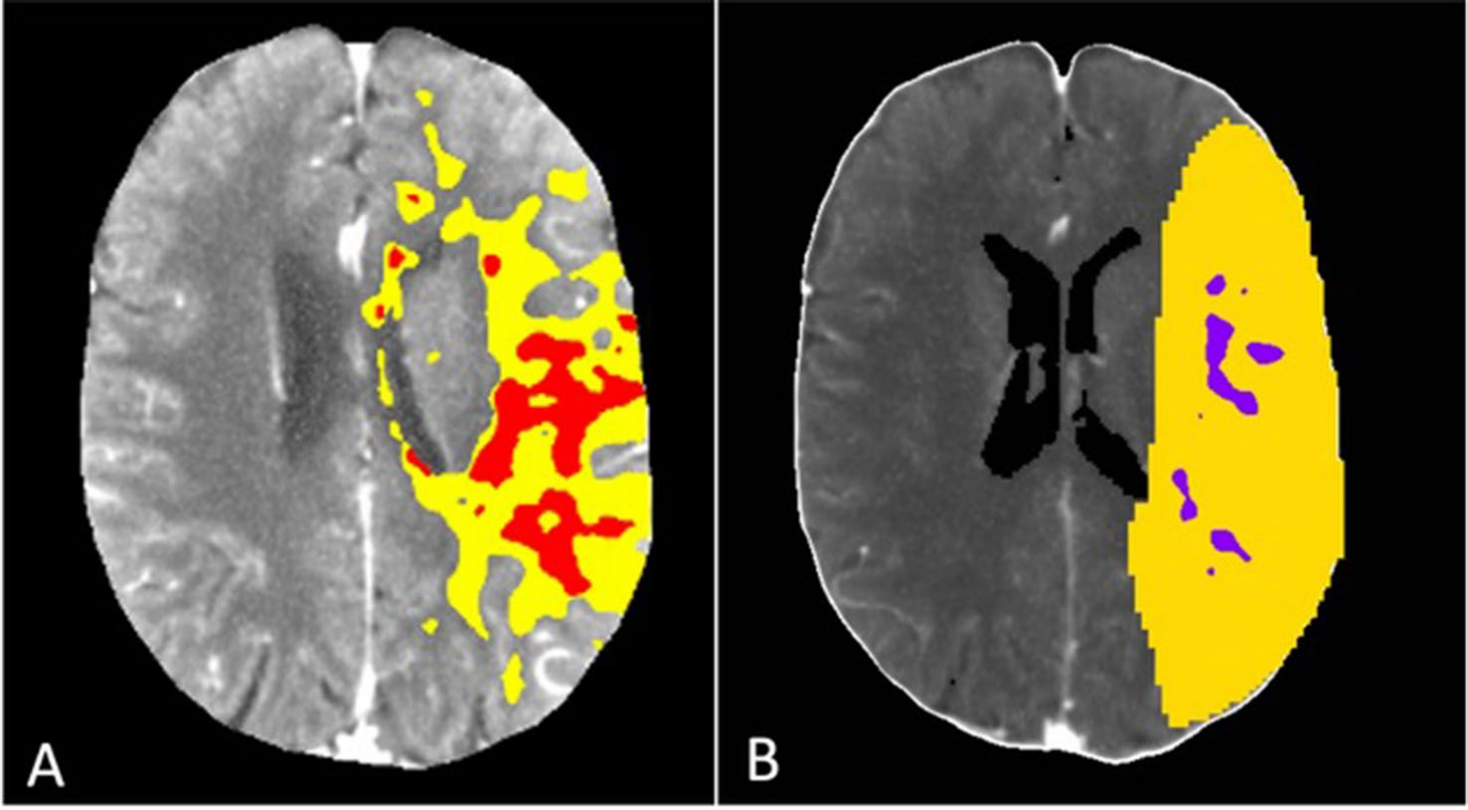
Illustrative summary maps for Syngo.via B (**A**) and Cercare (**B**) for a patient presenting with right-sided M1 occlusion. For syngo.via, infarct core is marked in red and penumbra in yellow, for Cercare infarct core is marked in violet and penumbra in yellow

The key baseline characteristics of the cohort were as follows: The median age was 76 years (IQR 66–82 years), the median baseline NIHSS score was 15. The median time from CTP to MT was 47 min (IQR 39–57 min) (Fig. 2).

### Segmented ischemic core volume

The median infarct volume for the included 65 patients was 7.3 ml (IQR 0.4–26.4 ml), with a mean of 28.1 ml (SD 50.6). The minimum infarct volume was 0 ml, the largest infarct volume was 273.0 ml.

### CT perfusion results

Of the 65 perfusion datasets, there was one study for each software (1.5%) that failed automated processing. For CMN, this was a right sided M1-occlusion with an ASPECTs of 5, NIHSS of 11, and CBF/CBV mismatch right parietal and in the basal ganglia. For all three syngo.via settings, a case of a proximal right-sided M1-occlusion with an ASPECTS of 8–9 and NIHSS of 20 could not be processed due to minor motion artifacts. Thus, final output was available for 64 patients (Table 1).

**Table 1:** Performance characteristics of CMN and syngo.via vs. the segmented true infarct volume

	Vol	CMN	syngo.via A	syngo.via B	syngo.via C
Median (IQR) infarct volume, ml	7.3 (0.4 to 26.4)	6.5 (1.5 to 20.5)	40.7 (28.8 to 56.0)	18.1 (10.7 to 31.0)	24.3 (11.1 to 37.8)
Min/Max infarct volume, ml	0.0 / 273.0	0.0 / 93.7	12.2 / 193.1	3.0 / 193.3	0.0 / 206.6
Mean (STD) infarct volume, ml	28.1 (50.6)	14.9 (20.2)	47.1 (30.4)	26.1 (27.9)	34.9 (36.0)
Infarct volume normal distribution (p-value)	0.000	0.000	0.000	0.000	0.000
Mean (SD) error, ml		-12.4 (40.4)	18.8 (40.9)	-2.1 (35.9)	6.6 (34.4)
Median (IQR) error, ml		0.0 (-14.0 to 6.3)	25.5 (7.9 to 38.6)	7.2 (-9.0 to 14.2)	8.1 (-1.5 to 26.8)
Limits of agreement, ml		-91.6, 66.9	-61.4, 99.0	-72.4, 68.1	-60.7, 74.0
ICC (95 % CI)		0.43 (0.21 to 0.61) (p-val: 0.000)	0.48 (0.24 to 0.66) (p-val: 0.000)	0.62 (0.45 to 0.75) (p-val: 0.000)	0.69 (0.54 to 0.80) (p-val: 0.000)
Pearson		0.654 (p-val: 0.000)	0.598 (p-val: 0.000)	0.737 (p-val: 0.000)	0.740 (p-val: 0.000)
Wilcoxon-Signed-Rank		863.000 (p-val: 0.236)	417.500 (p-val: 0.000)	832.000 (p-val: 0.164)	640.500 (p-val: 0.008)
Overestimation		32/64 (50.0%) Mean overestimation: 9.1 (8.1) Median overestimation: 6.3 (2.6 to 15.9) Mean underestimation: -34.9 (48.4) Median underestimation: -14.3 (-31.9 to -7.2)	52/64 (81.2%) Mean overestimation: 33.6 (23.4) Median overestimation: 29.3 (20.0 to 40.8) Mean underestimation: -45.0 (39.6) Median underestimation: -36.9 (-72.0 to -8.5)	44/64 (68.8%) Mean overestimation: 15.6 (12.6) Median overestimation: 10.8 (7.0 to 20.5) Mean underestimation: -41.2 (39.6) Median underestimation: -18.8 (-67.0 to -10.2)	47/64 (73.4%) Mean overestimation: 21.3 (19.7) Median overestimation: 15.3 (6.6 to 28.5) Mean underestimation: -34.0 (33.9) Median underestimation: -20.6 (-37.0 to -13.3)
Count:	65	64	64	64	64

**Fig. 3:**
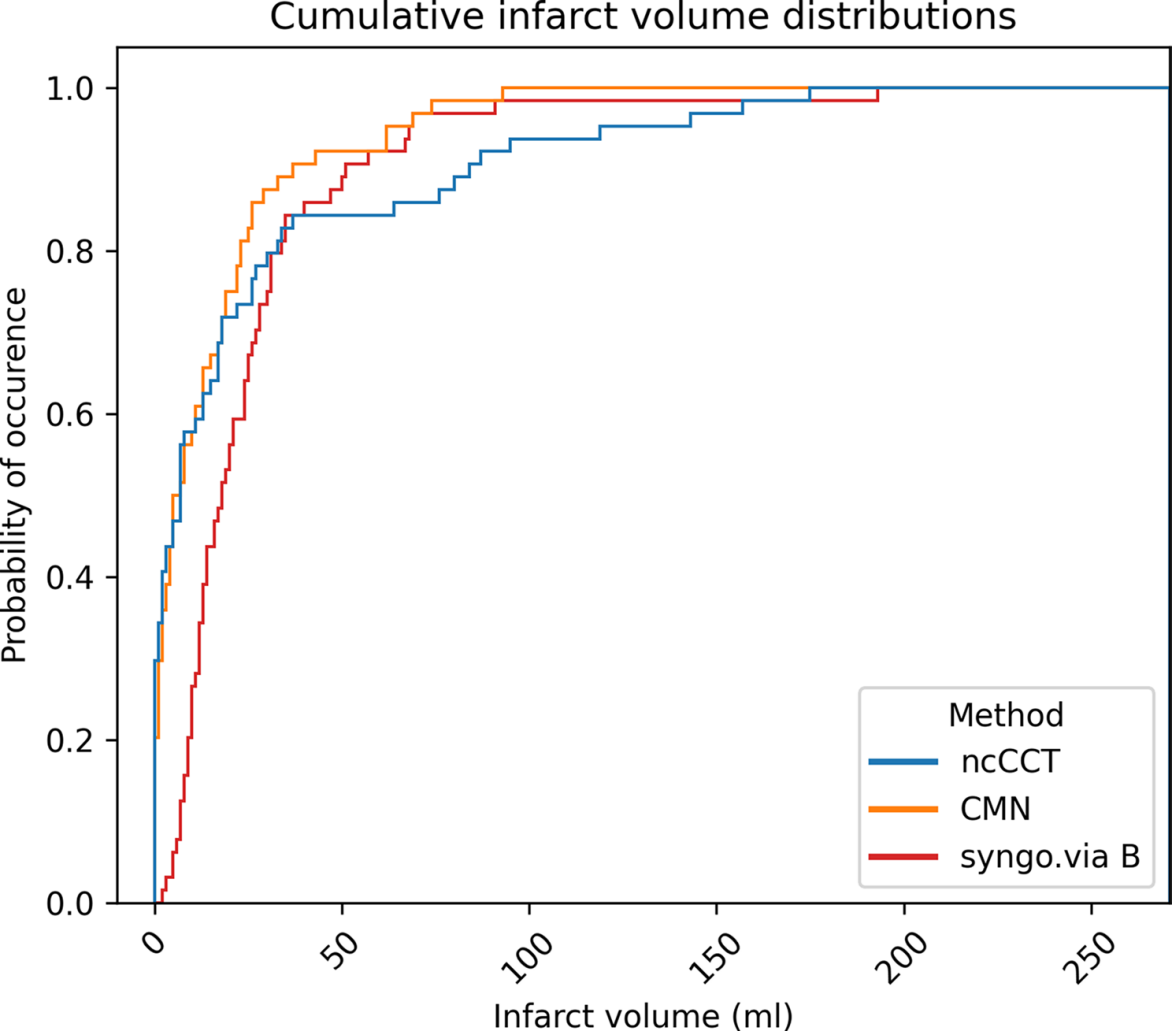
Histogram analysis of CMN vs. syngo.via B

Calculated median ischemic core volume by CMN was 6.5 ml (IQR 1.5–20.5), with a mean of 14.9 ml (SD 20.2 ml). The median error was 0.0 ml (IQR − 14.0–6.3 ml). The Wilcoxon-Signed-Rank test did not reveal relevant differences between the segmented volumes and the CMN output (*p* = 0.236). Overestimation occurred 32 of 64 patients (50.0%). For smaller infarcts, CMN showed a good match with ncCCT results in histogram analysis, with overestimations for larger infarct cores (Fig. 3).

Median ischemic core volumes for syngo.via were 40.7 (IQR 28.8–56.0) for setting A, 18.1 ml (IQR 10.7–31.0) for setting B, and 24.3 ml (IQR 11.1–37.8) for setting C. The core volumes were different between the segmented volume and setting A (*p* < 0.001) and setting C (*p* = 0.008). There was no relevant difference between setting B and the segmented volume (*p* = 0.164).

**Fig. 4:**
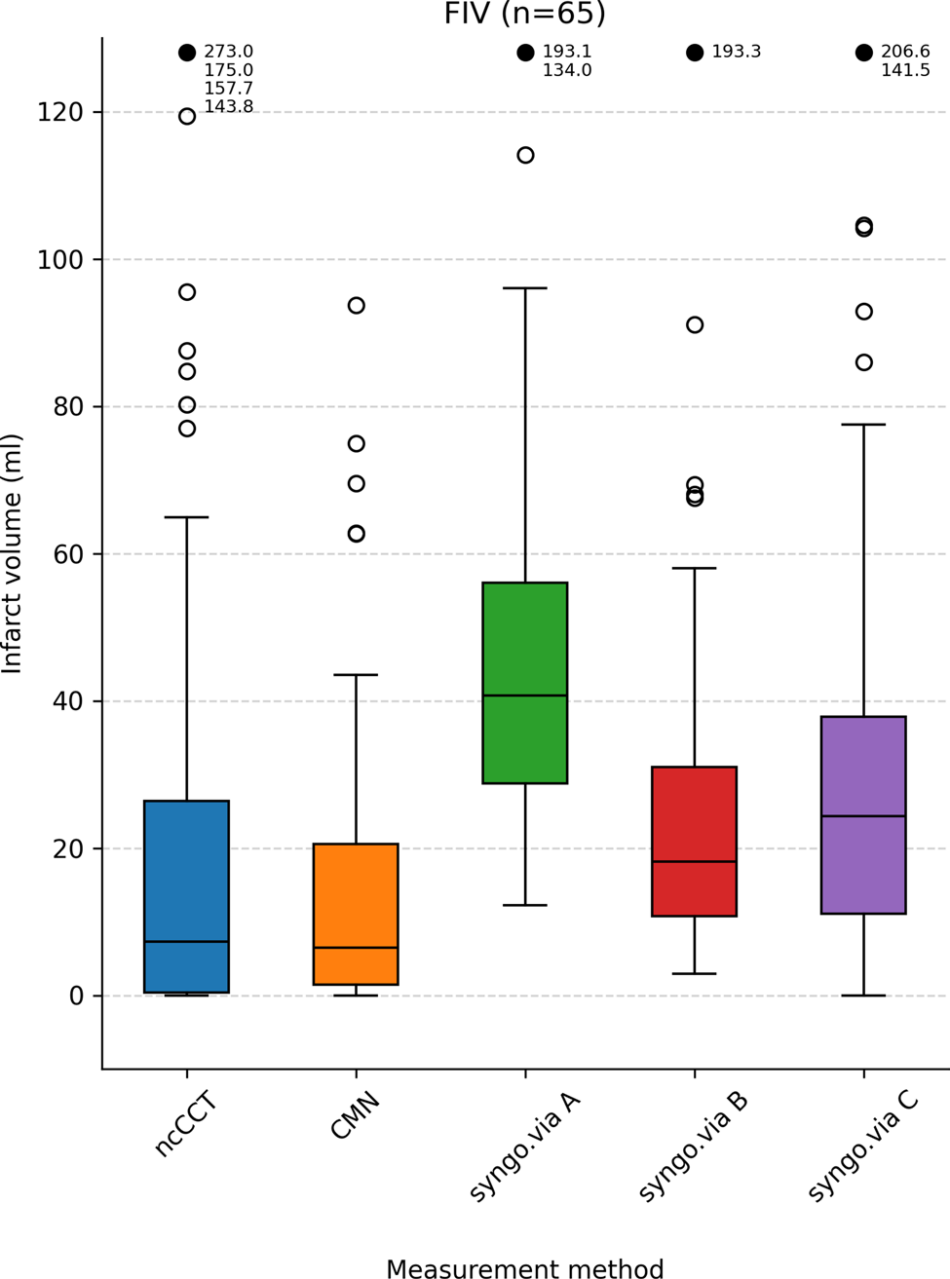
Boxplots illustrating agreement of CMN and syngo.via A-C with the true infarct volume

**Fig. 5:**
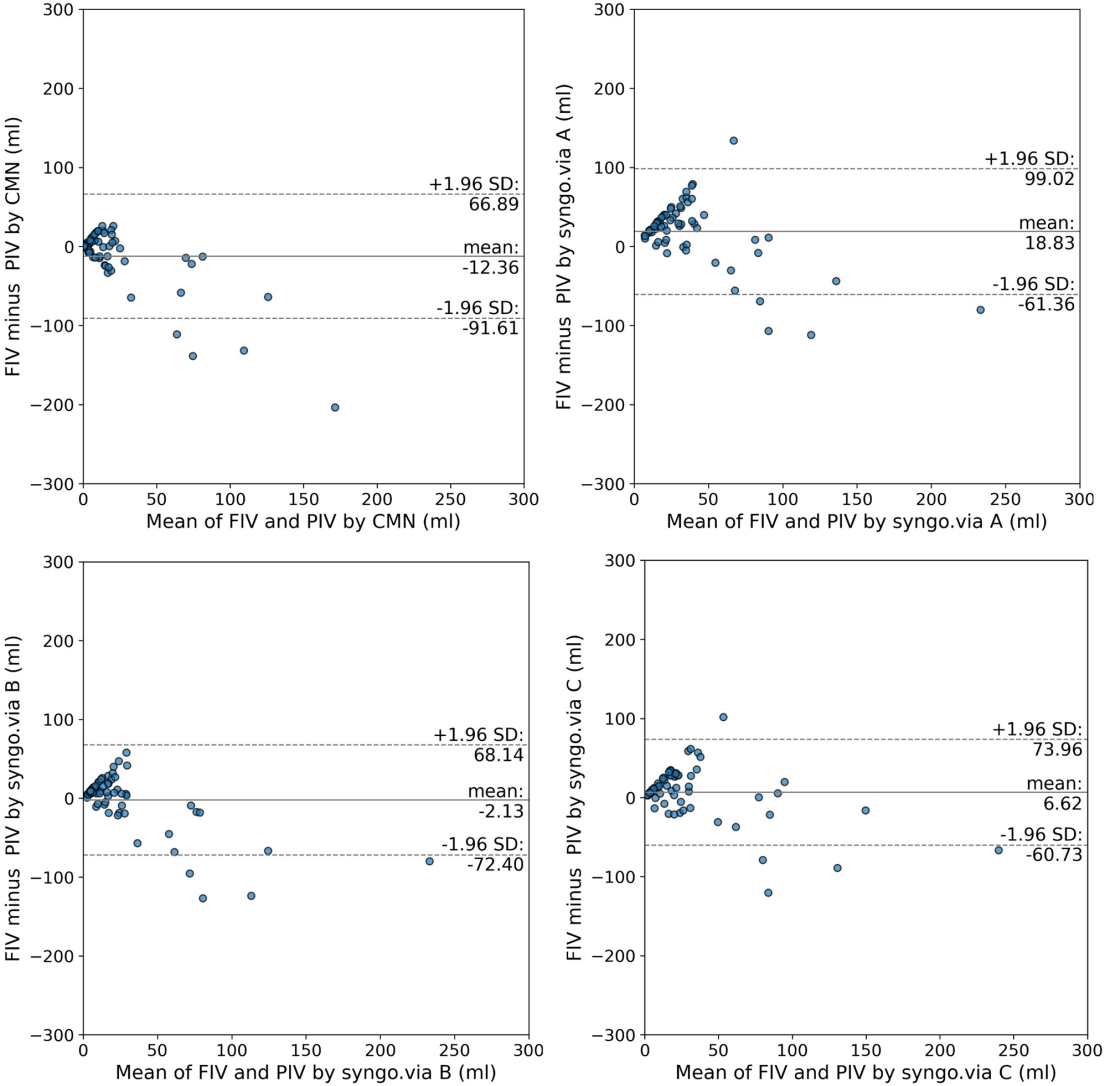
Bland-Altman plots for volumetric agreement of core volumes with CMN and syngo.via A-C. Solid lines indicate the mean difference between software outputs and infarct volume. Dotted lines represent 90% limits of agreement

The agreement between the segmented ischemic core volume and the CTP outputs are illustrated by boxplots in Fig. 4. The differences in relation to infarct core size are illustrated by Bland-Altman plots in Fig. 5. The mean (SD) difference in prediction errors between the segmented volume and syngo.via was 18.8 ml (SD 40.9 ml), -2.1 ml (SD 35.9 ml), and 6.6 ml (SD 34.4 ml) for settings A, B, and C, respectively. Comparison between CMN and the segmented volume showed a mean difference of -12.4 ml (SD 40.4 ml).

Limits of agreements for ischemic core were smallest for syngo.via setting B (-72.4, 68.1 ml) and largest for syngo.via setting A (-61.4, 99.0 ml).

**Fig. 6:**
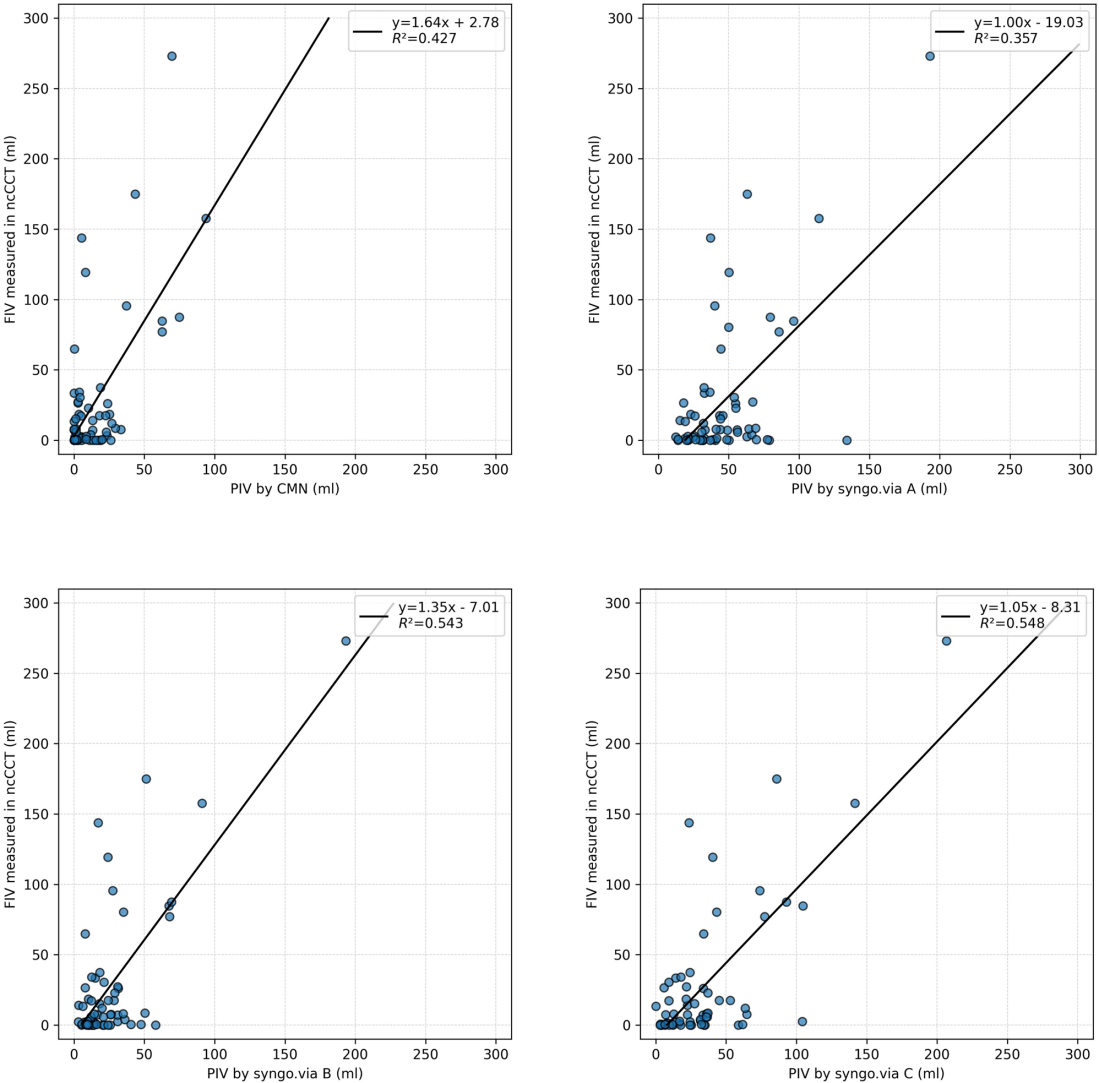
Scatterplots comparing core and penumbra outputs for CMN vs. syngo.via A-C

All software solutions exhibited a tendency to overestimate infarct volumes. Overestimation with syngo.via occurred in 81.2% for setting A, 68.8% for setting B, and 73.4% for setting C. Syngo.via B and C showed comparable correlations with final infarct volumes (Fig. 6), while syngo.via A showed the lowest.

**Fig. 7:**
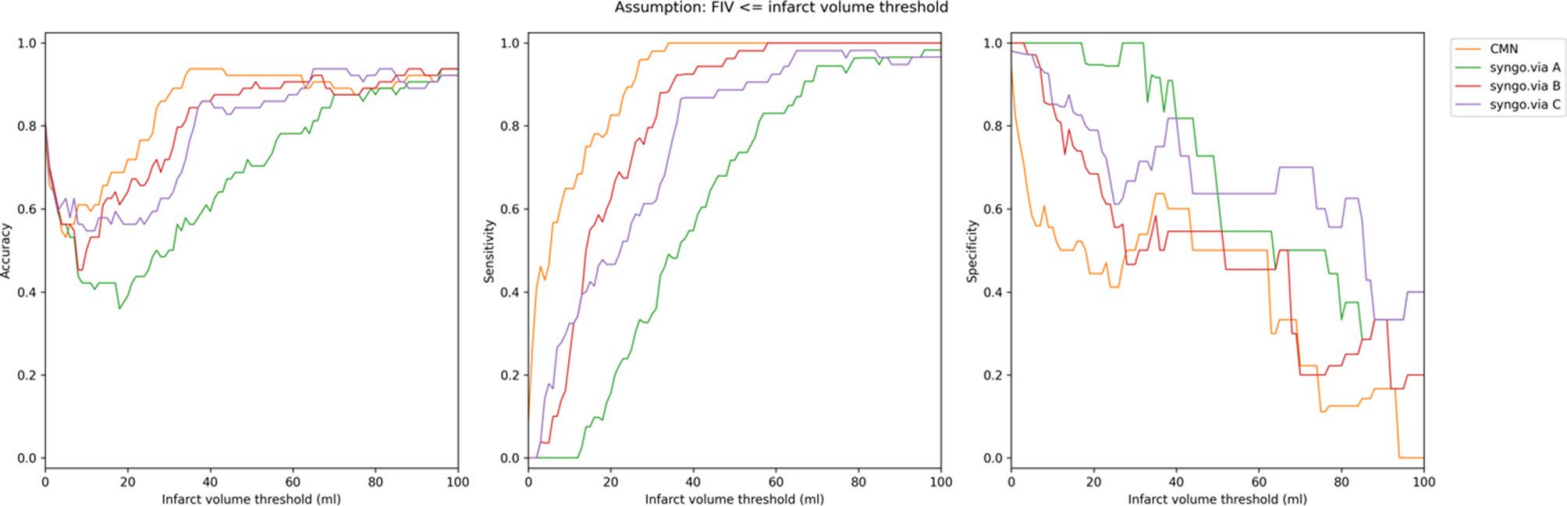
Accuracy, sensitivity and specificity analysis of CMN and syngo.via B

Figure 7 presents accuracy, sensitivity and specificity across infarct volume thresholds (0-100 ml). Accuracy for all methods was low at small infarct volumes. CMN and syngo.via B demonstrate the highest accuracy beyond 20 ml, with all methods converging toward approximately 90% accuracy for thresholds exceeding 60 ml. CMN showed the best sensitivity of all methods, achieving 100% sensitivity for infarcts above 60 ml, with syngo.via A showing the lowest sensitivity for all thresholds. Both CMN and syngo.via B show declining specificity for smaller infarct volumes, while syngo.via A maintains higher specificity for smaller thresholds.

## Discussion

In this study, we compared the accuracy of ischemic core volume estimates from Cercare Medical Neurosuite (CMN) and syngo.via (with three parameter settings) against true infarct volumes segmented on follow-up non-contrast CT. Overall agreement between software-generated core volumes and the actual infarct ranged from good to poor. CMN demonstrated generally good concordance with true infarct size, exhibiting the smallest median bias (0.0 mL) and the lowest frequency of overestimation among all methods. By contrast, syngo.via’s estimates tended to be larger than the true infarct, especially with its default parameter set. Notably, as the ischemic core volume increased, both CMN and syngo.via became less accurate, with their predictions deviating further from the actual infarct in patients with large strokes. This trend was evident in Bland–Altman analysis, where error magnitudes grew for larger cores, underscoring that mapping larger infarcts remains a challenge for current perfusion software. Clinicians should therefore interpret software-derived core volumes with caution in cases of large infarcts, as all methods showed some loss of accuracy at the upper volume range.

Across all comparisons, CMN performed better than syngo.via in key accuracy metrics. Syngo.via generally produced larger core volume estimates than CMN for the same patients, reflecting a tendency to overestimate infarct size. Applying syngo.via’s additional smoothing filter (setting B) did improve its agreement with true infarct volumes, consistent with prior reports [1]. In our results, CMN had a lower median error and fewer overestimations than syngo.via B, despite the latter showing the narrowest limits of agreement on Bland–Altman plots. The tighter limits of agreement for syngo.via B suggest that smoothing stabilized its estimates (particularly for large cores), yet CMN’s estimates were closer to the truth on average.

Syngo.via’s default setting (A, using a fixed CBV threshold of < 1.2 mL/100 mL) showed poor agreement, often substantially overestimating core size. The alternative syngo.via approach using an rCBF < 30% threshold (setting C) did not further improve accuracy in our cohort. In fact, the rCBF-based method frequently misjudged large infarcts – a pattern in line with a recent study by Hoving et al. where a 30% rCBF threshold led to significantly more frequent large-volume overestimations than other threshold approaches [3]. Thus, among the syngo.via configurations we tested, the smoothed CBV threshold (B) was the most reliable, yet CMN was superior to all syngo.via settings in terms of both bias and overestimation rate.

While CMN showed higher accuracy and sensitivity for smaller infarct volumes, syngo.via demonstrated comparable or even slightly superior performance in certain contexts. Specifically, syngo.via with smoothing (method B) exhibited the narrowest limits of agreement (-71.9 to 69.1 ml) in Bland–Altman analysis, indicating more consistent predictions across the entire spectrum of infarct volumes, especially at larger infarcts. While CMN showed lower overall median error and fewer overestimations, syngo.via B’s accuracy improved notably as infarct volumes increased, demonstrating reliability in detecting large ischemic cores. Additionally, syngo.via maintained relatively higher specificity at smaller infarct volume thresholds (< 20 ml). These results indicate that syngo.via, particularly with smoothing applied, can offer robust performance, especially in patients presenting with large infarcts or smaller borderline lesions.

We also observed that CMN delineated a smaller infarct core and a larger penumbra compared to syngo.via in each case, even though the total hypoperfused volume (core + penumbra) was similar between the two software. In practical terms, CMN identified more tissue as salvageable penumbra whereas syngo.via (especially without smoothing) often classified that tissue as already infarcted core. This difference suggests that CMN’s algorithm may better distinguish viable tissue from irreversibly infarcted tissue, particularly in the critical intermediate perfusion range. One possible reason may be CMN’s deconvolution method and incorporation of physiological parameters beyond simple thresholds. The CMN platform leverages models of tissue oxygenation and other perfusion dynamics to identify infarct core, which could yield more precise core–penumbra differentiation. In contrast, syngo.via relies on fixed threshold criteria (for CBV or relative CBF) that may not universally correspond to true tissue viability in all patients. This methodological difference may explain why CMN was better at predicting final infarct volume: its computational approach adapts to the perfusion state of tissue, whereas syngo.via’s threshold-based classification can mislabel moderately hypo-perfused (but ultimately salvageable) tissue as core, leading to overestimation.

Our findings are consistent with a growing body of literature demonstrating considerable variability in quantitative CTP outputs across different software solutions [19–21]. Previous comparative studies have similarly reported that estimated core volumes can differ substantially between post-processing platforms, even when applied to the same patient data [15, 20]. Koopman et al., for example, compared three perfusion software packages and found that syngo.via with smoothing (analogous to our setting B) yielded the closest agreement with their reference standard [1]. This aligns with our observation that syngo.via’s performance improved with the smoothing filter, though in our case the reference was the true infarct volume rather than another software.

On the other hand, some recent comparisons have noted instances of better concordance. Kim et al. observed remarkably high agreement between two automated CTP packages in estimating core and hypoperfused volumes within 24 h of stroke onset [2]. Likewise, a head-to-head study of Olea, MIStar, and syngo.via versus RAPID found only small differences in core volume outputs among these tools, even though perfusion lesion (penumbra) volumes varied more appreciably [22]. These reports suggest that when similar threshold criteria or deconvolution models are used, core volume estimates can converge.

In our study, however, the algorithms compared were quite different (proprietary AI-based vs. conventional thresholds), which likely magnified the divergence in results. We also intentionally applied each software in its default or recommended configuration rather than enforcing a uniform threshold across all. This contrasts with some prior studies that attempted to harmonize thresholds or calibrate each software against the final infarct. Muehlen et al. took such a threshold-tuning approach in a cohort of fully reperfused patients, identifying an optimal rCBF cutoff of roughly 30–34% for core definition in each software to best match final infarct on MRI [21]. Their approach highlights that the “best” threshold may vary by software; in fact, an rCBF < 30% (the default for RAPID and our syngo.via C) was slightly too conservative, tending to underestimate the true infarct volume unless raised closer to one-third of normal perfusion. By contrast, our methodology kept the vendors’ default settings (CBV < 1.2 for syngo A/B and rCBF < 30% for syngo C) and assessed performance without adjustment. This design decision underscores a unique aspect of our study: we included only patients with successful reperfusion (TICI 2c/3) and minimal delay to recanalization, thereby providing an ideal scenario in which the baseline CTP should predict final infarct if the software algorithms are accurate.

The role of AI in neuroimaging will likely expand. In CTP for acute stroke, current implementations primarily deploy AI at targeted steps, most notably motion correction and related preprocessing, while deconvolution remains the prevailing method for deriving perfusion variables [23]. In a recent review, Gragnano et al. underscore considerable inter-vendor variability in thresholds and output maps that can influence patient stratification, and they highlight persistent obstacles to broader AI integration, including black-box behavior, workflow challenges, and economic and regulatory constraints [23]. The review posits that integrating AI with clinical data could enable patient-specific CTP maps in the future and reduce reliance on rigid threshold-based decision making [23]. In this context, CMN’s proprietary approach aligns with this initial trajectory, using advanced modeling to refine perfusion analysis without discarding the underlying paradigm.

Our study excluded cases with incomplete recanalization to avoid confounding by infarct growth. Due to a short time to reperfusion, infarct growth was likely minimal, creating a valid case scenario for validating software performance. In line with these conditions, we observed that all software still showed a propensity to overestimate final infarct volumes to some degree (though least so with CMN). Notably, syngo.via’s default setting (A) overcalled the infarct in about 82% of cases in our series, and even the refined settings B and C were more often too high rather than too low in their estimates, compared to the actual infarct. While part of the discrepancy can be attributed to the slight infarct evolution that may occur even in the short window between imaging and reperfusion, our tight time frames and the selection of only TICI 2c/3 outcomes mean that infarct growth was likely minimal [24]. A software using a stricter threshold (rCBF 30% or a low CBV cut-off) will label less tissue as core at baseline – potentially missing some of the infarct – whereas a tool with a very lenient threshold might label too much as core (including penumbra). Our data suggest that CMN’s proprietary analysis has achieved a better balance in this trade-off under standard settings, whereas syngo.via’s default thresholds tend to err on the side of inclusion (yielding larger core volumes). Our findings are in line with a recent study by Broocks et al. who reported in a multicenter EVT cohort that baseline CTP-derived ischemic core overestimated the final infarct in 22% of patients, occurring more often when the core exceeded 50 mL and when reperfusion was successful [25]. In adjusted analyses, successful recanalization and larger baseline core volume independently predicted overestimation, whereas time from onset to imaging did not. These data reinforce that core calculations should be interpreted alongside software-specific bias when applying decision thresholds.

From a clinical standpoint, these findings have important implications for acute stroke management. Precise delineation of the infarct core and penumbra is crucial for making optimal treatment decisions [5]. Overestimation of core volume is particularly concerning because it can wrongfully exclude patients from potentially life-saving therapies. Our data suggest that software-related differences in core volume could alter treatment eligibility in borderline cases along DEFUSE 3 thresholds. This phenomenon has been described in earlier comparisons of perfusion software. For example, Bushnaq et al. found that RapidAI and Viz.ai produced systematically different core sizes [26]. While these differences did not significantly alter overall DAWN/DEFUSE-3 eligibility in their cohort, the authors, cautioned that individual patients might be classified differently by different platforms. Our results indicate that such a risk is higher with syngo.via’s outputs than with CMN. In practical terms, CMN’s lower tendency to overestimate means it is less likely to prematurely rule out patients who could benefit from reperfusion [20].

Certain limitations of our study must be acknowledged. First, this was a single-center study using one specific CT scanner and imaging protocol. Results might differ with other scanners or settings, as factors like signal-to-noise ratio and timing can affect perfusion analysis. However, recent evidence indicates that variability in CTP results stems mostly from differences in post-processing software rather than the scanner hardware [27]. Standardizing the perfusion software substantially harmonized results across different scanners. Thus, the discrepancies we observed are likely more attributable to software algorithms than to the imaging platform per se.

Second, we evaluated CMN using its standard, vendor-recommended settings. The software allows expert users to tweak thresholds or parameters, which we did not explore; such modifications might further improve accuracy or alter the results. Third, our ground truth for infarct size was the follow-up non-contrast CT segmentation at 24 h. While widely used, ncCCT is less sensitive than MRI DWI for detecting ischemic injury, especially for small cortical infarcts or early infarct changes. We mitigated potential ncCCT inaccuracies by having experienced readers perform the segmentation, but some error in final infarct volume measurement is possible. That said, any such error would affect all software comparisons equally and thus is unlikely to change the relative performance ranking. Fourth, the software versions we used were those available at the time of analysis; ongoing development means newer versions might behave differently. Finally, our sample size was modest, while comparable to similar analyses in the literature. This warrants caution in generalizing our results.

In conclusion, we found substantial variability in CTP-derived core volume estimates between CMN and syngo.via. In our successfully reperfused patient cohort, CMN and syngo.via (with smoothing) showed the best agreement with final infarct volumes, whereas syngo.via’s default and rCBF-threshold settings were prone to notably greater overestimation. CMN emerged as the most accurate tool overall, achieving the closest match to true infarct size and the lowest rate of overestimating the core. These results underscore that different software platforms – and even different parameter settings within the same platform – can yield markedly different perfusion results. Such differences should be acknowledged by clinicians when interpreting perfusion maps, particularly for large infarcts where the discrepancies are most pronounced. Going forward, efforts to standardize perfusion analysis or incorporate advanced physiological modeling may further improve accuracy. Understanding strengths and weaknesses of software solutions enables clinicians to better judge the reliability of the data at hand and ultimately to optimize care for patients.

## Data Availability

The raw data supporting the conclusions of this article will be made available by the authors upon reasonable request.

## Abbreviations

AIS: Acute ischemic stroke
ASPECTS: Alberta Stroke Program Early CT Score
CBV: Cerebral blood volume
CBF: Cerebral blood flow
CMN: Cercare Medical Neurosuite
CT: Computed tomography
CTP: Computed tomography perfusion
EVT: Endovascular thrombectomy
ICC: Intraclass correlation coefficient
IQR: Interquartile range
MCA: Middle cerebral artery
MT: Mechanical thrombectomy
mTICI: Modified Thrombolysis in Cerebral Infarction
ncCCT: Non-contrast computed tomography
NIHSS: National Institutes of Health Stroke Scale
rCBF: Relative cerebral blood flow
SD: Standard deviation
SVD: Singular value decomposition
TICI: Thrombolysis in Cerebral Infarction
